# Supplementary material for: Gender dynamics in the relationship among student-teacher relationships, academic task engagement, academic task value, and positive developmental outcomes
Source: PLoS One. 2025 Dec 31;20(12):e0340112. doi: 10.1371/journal.pone.0340112 (PMC12755818; doi:10.1371/journal.pone.0340112)
Supplement: Attachment — Submitted filename: Ethical Clerance.pdf [file pone.0340112.s004.pdf]

Bahir Dar University
ባህር ዳር ዩኒቨርሲቲ
College of Education
የትምህርት ኮሌጅ

Institutional Research Review Committee (IRRC)
IRRC'S Decision

Meeting No. 6 / 24 **Date(D/M/Y):** 23/09/2024
Protocol number: 001954 **Assigned No:** 011

**Protocol title:** Contributions of Family Functioning and Classroom Contexts on Promoting Positive Development among Bahir Dar Secondary School Students'							
**Name of Principal investigator:** Getahun Tadesse Abren<br>**Institution of principal investigator:** Bahir Dar University							
**Version of elements reviewed**	Protocol version:	2	Attached: yes	√	No		
	Consent form version:	2	Attached: yes	√	No		
**Decision of the board:**	Approved	√	Resubmission		Disapproved		
	Sent to National Ethics Review Committee	√					

Obligations of the Principal investigator:

1. Complies with the standard international & national scientific and ethical guidelines
2. Gets IRB approval for all amendments and changes required to be made in the protocol and consent form
3. Reports SAE within 10 days of the event
4. Report send of the study, including manuscripts and thesis works
5. Reports non-compliance and unanticipated events

Approval Period (23rd Sep./2024-22nd Sep. /2025)

**Follow up report expected in:**	3months		6months	
	9months		12months	√

Jerusalem Yibeltal Yizengaw (PhD)
Assistant Professor of International & Comparative Education
Chair Person, IRRC
Signature [signature]
Date 23/09/2024

Bahir Dar University College of Education
Email: BDUCOEIRRC@bdu.edu.et
Institutional Research Review Committee (COEIRRC)
