# Supplementary material for: Gender dynamics in the relationship among student-teacher relationships, academic task engagement, academic task value, and positive developmental outcomes
Source: PLoS One. 2025 Dec 31;20(12):e0340112. doi: 10.1371/journal.pone.0340112 (PMC12755818; doi:10.1371/journal.pone.0340112)
Supplement: Attachment — Submitted filename: Gender dynamics Review Report.docx [file pone.0340112.s002.docx]

**Dear Editor and Authors,**

**I appreciate the opportunity to read the manuscript titled "Gender dynamics in the relationship among student-teacher relationships, academic task engagement, academic task value, and positive developmental outcomes." The study is well-targeted to a crucial and relevant educational psychology problem employing a good methodological strategy to examine gender-differentiated pathways. The findings have helpful implications for educationally gender-sensitive practice. However, several of the points require clarification and elaboration to enhance the contribution and scholarship of the manuscript.**

**Abstract: Overall, the abstract is a useful summary of the principal quantitative findings but is too-statistical. Including an introductory sentence positioning the significance of the study would be useful. The last sentence on implications is excellent but slightly too general. Throughout, use wording that keeps the correlational nature of findings (e.g., "were associated with" instead of "account for").**

**Introduction: The introduction is very clearly establishing the importance of the central constructs. The link between perceived academic task value and positive developmental outcomes is only suggested, however, and might be more directly theorized. Although international literature is cited, the presence of a specific theoretical model (e.g., Expectancy-Value Theory) more centrally would provide stronger rationale for hypothesizing the mediated relationships. The research question is well-stated.**

**Methods: The methodology in general is strong but has several details to sort through:**

**-Sampling: The multi-stage sampling method is clearly explained. Could you, however, specify how the 13 classrooms were chosen among the five schools (e.g., random selection, all five schools' classrooms?) and give the final response rate of the participants after the exclusion of incomplete responses?**

**-Measures: The process of cultural and linguistic adaptation of the instruments is explained superbly and is a key strength. The psychometric properties included (CFA, Cronbach's alpha) are strong.**

**-Analysis: Usage of MGSEM is very appropriate. A tragic error is in criteria for fit index published. CMIN/DF < .50 has been mentioned to show good fit. This is incorrect and unrealistically strict. The accepted values are CMIN/DF < 3 or < 5. This should be revised to avoid misinterpretation, even though the reported figures in themselves are all within the acceptable limit (< 3).**

**-Data Availability: The statement is short and true.**

**Results:**

**-Descriptive Statistics (Table 1): The statement is females reported higher mean scores across all constructs. This important finding needs to be supported by presenting results of independent samples t-tests (t-values and p-values) to ensure that these differences are statistically significant.**

**-Measurement Invariance (Table 3): Reaching scalar invariance is a great strength, and it attests to the use of MGSEM.**

**-Direct Effects (Table 5): Well presented. The nuances of the gender differences in paths (e.g., STR for male behavioral involvement stronger, ATV for female behavioral involvement stronger) are compelling and well-highlighted.**

**-Indirect Effects (Table 6): Typo in the estimate for the female "STR to PD via BEH" (40% CI to 95% CI). These mediated paths are easy to interpret.**

**Discussion: The discussion sufficiently captures the results in the present literature. To make the analysis more profound, observe:**

**-Briefly mention how the distinctive educational and cultural environment of Ethiopia can assist in explaining observed gender trends. This would enhance the globalizability of the results.**

**-The limitation concerning self-report information is understandable. Cross-sectional design must also be explicitly stated as a limitation because it precludes causation inference.**

**-Practical teacher implications are well placed. A sentence or two regarding implications for teacher training programmes or school policy will help to maximize the scope.**

**-In general, the manuscript is very well written. Some minor proofread and grammar adjustments are proposed (e.g., Page 11, line 107: "These students' positive developmental outcomes" -> "Positive developmental outcomes"). Ensure that all acronyms are defined at first usage (e.g., PYD on page 14). Tables are legible but ensure that all the decimal places are consistent.**

**References:**

**-The reference list is complete and usable. Reference #6 ("Rey Educ Res. 2011.") appears to be incorrectly referenced because the list of authors is not present. This needs to be edited to "Roorda, D. L., et al. (2011).".**

**- The literature review is strong. To further cite the study, cite the conceptual theoretical work on the Five Cs model of Positive Youth Development (e.g., Lerner et al., 2011, Journal of Youth and Adolescence) over the reference for the measurement tool.**
