# Supplementary material for: Gender dynamics in the relationship among student-teacher relationships, academic task engagement, academic task value, and positive developmental outcomes
Source: PLoS One. 2025 Dec 31;20(12):e0340112. doi: 10.1371/journal.pone.0340112 (PMC12755818; doi:10.1371/journal.pone.0340112)
Supplement: Attachment — Submitted filename: Rview report PLos.docx [file pone.0340112.s003.docx]

This is a well-designed and executed study that addresses a significant and timely research gap. The application of Multi-Group Structural Equation Modeling (MGSEM) to explore gender-specific pathways in an Ethiopian context is a notable strength. The manuscript is generally well-written, the methodology is sound and transparent, and the findings have clear, practical implications for educational practice. It is a strong candidate for publication after addressing the points below.

**Major Points for Consideration**

1. **Sampling and Representation:**

The sample is heavily skewed towards female students (59.2% final sample). While this can happen in random sampling, it should be briefly acknowledged in the limitations section. A comment on whether this proportion is representative of the general student population in Bahir Dar would be useful.

The **average age** of participants is provided (16.69), but the **age range** would be helpful context.

2. **Clarity in Results Reporting:**

**Table 4 (R² values):** The p-values for all R² estimates are listed as .001 or .002. However, R² is a measure of variance explained, and its significance is typically inferred from the overall model fit and path significances. Reporting a p-value for R² itself is unusual. Please confirm the statistical test being reported here.

**Page 27, Indirect Effects:** The text states: "females a coefficient of (β = 0.086, **40% CI** [0.036, 0.135])". This appears to be a typo and should read "**95% CI**".

**Page 28, Table 6:** The estimates for "Total Indirect Effect of STR" and "Total Indirect Effect of ATV" are listed as 0.000 for both estimate and CI, which seems implausible given the specific indirect effects listed above are not zero. This likely requires correction.

3. **Discussion Nuance:**

The discussion could briefly speculate on the *cultural reasons* behind the observed gender differences within the Ethiopian context. Why might teacher relationships be more salient for boys? Why might task value be a stronger driver for girls? Adding a sentence or two would enrich the interpretation.

When citing references in the discussion, ensure they perfectly align with the point being made. For example, on Page 30, the citation [33] is about "school-based collaboration," which seems less directly relevant to the point about motivational interventions than other potential citations might be.

**Minor comments**

**Required Editorial Corrections**

1. **Abstract:**

The beta (β) symbols in the abstract are currently used to represent R² (variance explained). This is statistically incorrect. Beta (β) represents standardized regression coefficients. The abstract should state "R² = 0.403" instead of "β = 0.403". This error appears throughout the abstract. **This is a critical correction.**

2. **Data Availability Statement:**

The statement is currently appropriate for restricted data. However, ensure the journal's policy is met. The text "The datasets that support the conclusions of this study are available from the corresponding author upon reasonable request" is standard and acceptable.

3. **Formatting and Typos:**

**Page 8, Line 31-32:** 95% CI [0.279, 0.516], p < .001), while they explain 31.9% of the variance for female students (β = 0.319, 95% CI [0.224, 0.408], p < .001). -> Again, these should be R² values, not β.

**Page 13, Line 143:** Bahir Dar City Administration, which includes a total of 21 secondary schools (– i.e., 11 public and 10 private) -> The format "(– i.e., ...)" is awkward. Use "(i.e., 11 public and 10 private)".

**Page 17, Line 239:** 5 point Lickert scale -> Should be "**5-point Likert scale**".

**Page 21, Table 1:** The last variable is labeled "Positive engagement (PD)" but should be "**Positive development (PD)**" to match the text and construct.

**Page 25, Table 5:** The column header C.R. is defined as "Critical ratio" in the note. It is more commonly referred to as the "Critical Ratio" (which is equivalent to a z-score for testing parameter estimates). This is fine, but ensure consistency.

**Page 30, Line 462:** had significa3nt positive effects -> Typo: "**significant**".

4. **Reference Consistency:**

**Page 36, Ref 6:** Rey Educ Res. 2011; -> Typo: Should be "**Rev Educ Res.**"

Please perform a final thorough check to ensure all in-text citations have a corresponding entry in the reference list and that all references are correctly formatted according to PLOS ONE guidelines.
