# Supplementary material for: Gender dynamics in the relationship among student-teacher relationships, academic task engagement, academic task value, and positive developmental outcomes
Source: PLoS One. 2025 Dec 31;20(12):e0340112. doi: 10.1371/journal.pone.0340112 (PMC12755818; doi:10.1371/journal.pone.0340112)
Supplement: Attachment — Submitted filename: Plos Reviewer report.docx [file pone.0340112.s005.docx]

This is a well-structured, methodologically sound, and theoretically grounded study that addresses an important and under-researched topic—gender differences in educational processes and outcomes in an Ethiopian context. The use of Multi-Group Structural Equation Modeling (MGSEM) is appropriate and rigorous. The manuscript is clearly written, and the findings have meaningful implications for educational practice and policy. I recommend acceptance with minor revisions.

**Minor Concerns and Suggestions for Revision**

**1. Abstract**

The abstract is clear but could be slightly more accessible to a broader audience. Consider rephrasing statistical results (e.g., “account for” → “were associated with”) to better reflect the correlational design.

**2. Introduction**

The link between **academic task value** and **positive developmental outcomes** could be more explicitly theorized. Consider strengthening the theoretical justification using Expectancy–Value Theory.

**3. Methods**

**Sampling**: Clarify how the 13 classrooms were selected (e.g., random lottery from all Grade 9–10 classrooms in the five schools).

**Model Fit Indices**: The manuscript correctly reports CMIN/DF < 5 as acceptable, but earlier versions contained an error (CMIN/DF < 0.50). Ensure this correction is consistent throughout.

**4. Results**

**Table 1 (Descriptive Statistics)**: Include results of independent samples t-tests (t-values, p-values) to support claims of gender differences.

**R² Reporting**: Ensure that all references to R² are correctly labeled (not as β) and that p-values are not incorrectly associated with R².

**Confidence Intervals**: Correct minor typos (e.g., “40% CI” → “95% CI”) in indirect effects reporting.

**5. Discussion**

**Cultural Context**: Briefly speculate on how Ethiopian cultural norms (e.g., gender roles, educational values) may explain the observed gender differences.

**Citation Alignment**: Ensure all citations directly support the claims made (e.g., replace [33] with a more relevant reference if needed).

**6. Limitations**

Explicitly acknowledge the **cross-sectional design** as a limitation for causal inference.

Note the **gender imbalance** in the sample (59.2% female), but clarify that this reflects the actual student population in Bahir Dar.

**7. References**

Ensure all references are complete and correctly formatted (e.g., Reference #6 should include full author list: Roorda et al., 2011).

Prefer citing foundational theoretical works (e.g., Lerner et al., 2005, 2011) over measurement tool references when discussing the Five Cs model.

**Editorial and Formatting Issues**

Correct minor typographical errors (e.g., “Lickert” → “Likert”, “significant” → “significant”).

Ensure all acronyms are defined at first use (e.g., PYD on page 14).

Standardize decimal places and formatting across tables.
