# Supplementary material for: Gender dynamics in the relationship among student-teacher relationships, academic task engagement, academic task value, and positive developmental outcomes
Source: PLoS One. 2025 Dec 31;20(12):e0340112. doi: 10.1371/journal.pone.0340112 (PMC12755818; doi:10.1371/journal.pone.0340112)
Supplement: Attachment — Submitted filename: Response to Reviewers.docx [file pone.0340112.s006.docx]

**Response to Reviewers**

**Subject:** Responses to Comments from Reviewers

**Manuscript Title:** Gender dynamics in the relationship among student-teacher relationships, academic task engagement, academic task value, and positive developmental outcomes

**Journal:** PLOS ONE

**Manuscript ID:** PONE-D-25-33636R1

**Dear Xiaopeng Wu and Reviewers,**

We appreciate the constructive feedback provided by the reviewers and the academic editor. We are carefully revising the manuscript to address all the comments and suggestions to ensure it fully meets PLOS ONE’s publication criteria. We will provide a detailed point-by-point response to each reviewer’s comment along with the revised manuscript upon resubmission.

| **Reviewer’s Comments** | **Authors Response** |
| --- | --- |
| 1. **Abstract**  - The abstract is clear but could be slightly more accessible to a broader audience. Consider rephrasing statistical results (e.g., “account for” → “were associated with”) to better reflect the correlational design. | - Thank you for this helpful observation. We have revised the abstract to enhance accessibility for a broader audience and to ensure the wording accurately reflects the correlational nature of the study. Specifically, statistical terms such as “account for” have been replaced with phrasing like “were associated with” to avoid implying causation. These modifications aim to improve clarity while maintaining the integrity of the findings. |
| 2. **Introduction**  - The link between **academic task value** and **positive developmental outcomes** could be more explicitly theorized. Consider strengthening the theoretical justification using Expectancy–Value Theory. | - Thank you for highlighting the need to further articulate the theoretical link between academic task value and positive developmental outcomes. In response, we have strengthened the theoretical justification in the Introduction by integrating key principles from Expectancy–Value Theory. Specifically, we incorporated foundational literature demonstrating how students’ task value beliefs shape engagement, persistence, and achievement-related behaviors (e.g., Wigfield and Eccles 2000; Eccles et al. 1983; Eccles and Wigfield 2002). These works clarify how valuing academic tasks can support broader developmental competencies, thereby providing a more explicit conceptual pathway connecting academic task value to positive youth development. The revised section now presents a clearer and more theoretically grounded rationale for examining this relationship. |
| 3. **Methods**  - **Sampling**: Clarify how the 13 classrooms were selected (e.g., random lottery from all Grade 9–10 classrooms in the five schools). - **Model Fit Indices**: The manuscript correctly reports CMIN/DF < 5 as acceptable, but earlier versions contained an error (CMIN/DF < 0.50). Ensure this correction is consistent throughout. | - Thank you for this constructive suggestion. We have revised the Sampling section to provide a more detailed explanation of how the 13 classrooms were selected. Specifically, we now clarify that after randomly selecting five schools (three public and two private), a lottery method was used to randomly select 13 classrooms from Grades 9 and 10 within these schools. This addition improves transparency and strengthens the methodological description of the sampling procedure. - Thank you for pointing out this inconsistency. We have carefully reviewed the manuscript and corrected all instances where the model fit criterion for CMIN/DF was previously misstated as “< 0.50.” The text now consistently reflects the appropriate threshold (CMIN/DF < 5) throughout the manuscript. This ensures accuracy and alignment with established SEM fit index guidelines. |
| 4. **Results**  - **Table 1 (Descriptive Statistics)**: Include results of independent samples t-tests (t-values, p-values) to support claims of gender differences. - **R² Reporting**: Ensure that all references to R² are correctly labeled (not as β) and that p-values are not incorrectly associated with R². - **Confidence Intervals**: Correct minor typos (e.g., “40% CI” → “95% CI”) in indirect effects reporting. | - Thank you for this helpful recommendation. To address your comment, we have added a new table (Table 2) presenting the independent samples t-test results, including t-values and p-values, to empirically support the statements regarding gender differences. This addition strengthens the descriptive section and provides clearer statistical evidence for the reported group comparisons. - Thank you for noting this issue. We have carefully reviewed all sections of the manuscript in which R² values are reported. Any instances where R² was mistakenly labeled as β have been corrected, and all incorrect associations of p-values with R² have been removed. The revised manuscript now consistently presents R² values accurately and in accordance with standard reporting conventions. - Thank you for drawing attention to these typographical errors. We have carefully reviewed the indirect effects section and corrected all incorrect confidence interval labels, including replacing “40% CI” with the appropriate “95% CI.” The revised manuscript now accurately reflects the correct confidence interval notation throughout. |
| 5. **Discussion**  - **Cultural Context**: Briefly speculate on how Ethiopian cultural norms (e.g., gender roles, educational values) may explain the observed gender differences. - **Citation Alignment**: Ensure all citations directly support the claims made (e.g., replace [33] with a more relevant reference if needed). | - Thank you for this valuable suggestion. In the revised Discussion section, we have incorporated a brief consideration of how Ethiopian cultural norms may help explain the observed gender differences. Specifically, we discuss how traditional gender role expectations, societal views on education, and cultural emphasis on diligence and responsibility may shape boys’ and girls’ engagement patterns. For example, girls in Ethiopia may experience stronger links between perceived task value and behavioral engagement due to cultural encouragement toward persistence and academic diligence, whereas boys may rely more on relational support from teachers because of social norms that afford them greater autonomy. These culturally grounded interpretations complement the existing theoretical and empirical discussion, providing context-specific insight while remaining consistent with the correlational nature of the study’s findings. - Thank you for emphasizing the importance of aligning citations with the claims presented. In the revised manuscript, we reviewed the earlier citation and confirmed that it did not adequately support the argument. Accordingly, we replaced it with a more relevant and empirically aligned source. In the current version of the manuscript, this updated citation appears as reference [36]: Lesperance et al. (2022), which more appropriately addresses gender differences in motivational–affective factors. This correction ensures that the citation now fully supports the claim and maintains coherence within the revised reference list. |
| 6. **Limitations**  - Explicitly acknowledge the **cross-sectional design** as a limitation for causal inference. - Note the **gender imbalance** in the sample (59.2% female), but clarify that this reflects the actual student population in Bahir Dar. | - Thank you for these important observations. In the revised manuscript, we explicitly acknowledge that the cross-sectional design limits the ability to draw causal inferences from the observed relationships. Additionally, we note the gender imbalance in the sample (59.2% female) and clarify that this proportion accurately reflects the actual student population in Bahir Dar. These clarifications enhance transparency regarding the study’s methodological constraints while contextualizing the sample characteristics. |
| 7. **References**   Ensure all references are complete and correctly formatted (e.g., Reference #6 should include full author list: Roorda et al., 2011).  Prefer citing foundational theoretical works (e.g., Lerner et al., 2005, 2011) over measurement tool references when discussing the Five Cs model. | - Thank you for highlighting this issue. We have carefully reviewed all references and ensured that they are complete and correctly formatted according to journal guidelines. Specifically, Reference #6 has been updated to include the full author list and accurate bibliographic details: Roorda DL, Koomen HMY, Spilt JL, Oort FJ (2011). The influence of affective teacher–student relationships on students’ school engagement and achievement: a meta-analytic approach. Review of Educational Research, 81(4), 493–529. <https://doi.org/10.3102/0034654311421793> - Thank you for this valuable suggestion. In the revised manuscript, we have prioritized citing foundational theoretical works when discussing the Five Cs model of Positive Youth Development. Specifically, we now reference Lerner et al. (2005, 2011), which provide the conceptual basis for the model, rather than measurement-focused sources. These citations more accurately support the theoretical discussion and contextualize the Five Cs within the broader PYD framework:   [15] Lerner RM, Almerigi JB, Theokas C, Lerner JV. (2005). Positive youth development: A view of the issues. Journal of Early Adolescence, 25(1), 10–16. https://doi.org/10.1177/0272431604273211  [16] Lerner RM, Lerner JV, Lewin-Bizan S, Bowers EP, Boyd MJ, Mueller MK, et al. (2011). Positive youth development: Processes, programs, and problematics. Journal of Youth Development, 6(3), 38–62. https://doi.org/10.5195/JYD.2011.174  This change ensures the discussion emphasizes the theoretical underpinnings of the Five Cs model. |
| **Editorial and Formatting Issues**  Correct minor typographical errors (e.g., “Lickert” → “Likert”, “significant” → “significant”).  Ensure all acronyms are defined at first use (e.g., PYD on page 14).  Standardize decimal places and formatting across tables. | Thank you for these detailed suggestions. We have carefully reviewed the manuscript and implemented the following corrections:   - Minor typographical errors have been corrected (e.g., “Lickert” → “Likert”). - All acronyms are now defined at first use, including PYD on page 14. - Decimal places and numerical formatting have been standardized across all tables to ensure consistency and clarity.   These revisions improve the manuscript’s readability, accuracy, and professional presentation. |

***Final Remarks:*** We sincerely appreciate the thoughtful and constructive feedback provided by the reviewers and the academic editor. Their insights have been instrumental in refining and strengthening the manuscript. In this revision, we have enhanced the clarity of the narrative, expanded the theoretical justification, provided additional methodological details, corrected reporting inconsistencies, and refined the presentation of results. We have also addressed all noted limitations, ensured citation accuracy, improved reference formatting, and corrected minor typographical and formatting issues throughout. We believe these revisions have substantially improved the quality and rigor of the manuscript.

Sincerely yours,

Authors
